# Analysis of influencing factors and construction of prediction model for multidrug-resistant tuberculosis in Nanning area

**DOI:** 10.3389/fpubh.2025.1599578

**Published:** 2025-12-03

**Authors:** Jie Huang, Qing-dong Zhu, Kan Xie, Ting-ting Lu, Xing-fa Lu, Jie-ling Chen, Hai-ling Yu, Yan-ling Hu

**Affiliations:** 1 Faculty of Data Science, City University of Macau, Macau, China; 2 HIV/AIDS Clinical Treatment Center of Guangxi (Nanning) and The Fourth People’s Hospital of Nanning, Guangxi, China

**Keywords:** Mycobacterium, tuberculosis, drug resistance, receiver operating characteristic, predictive model

## Abstract

**Objective:**

This study aims to analyze the characteristics of multidrug-resistant *Mycobacterium tuberculosis* isolates and to identify the factors influencing multidrug resistance in the Nanning area.

**Methods:**

This study retrospectively analyzed all sputum specimens from pulmonary tuberculosis patients collected at the Fourth People’s Hospital of Nanning from January 2021 to June 2022, including a total of 337 strains of *Mycobacterium tuberculosis*. Univariate analysis and binary logistics regression analysis were used to identify factors influencing multidrug resistance. A predictive model was constructed with SPSS software, and the predictive value of the model was evaluated with the Receiver Operating Characteristic (ROC) curve.

**Results:**

The results of binary logistics regression analysis indicated that treatment status and high-risk population were independent factors influencing multidrug resistance (*p* < 0.05). According to the logistics regression analysis results, the model was constructed as follows: Logit(P) = −1.874 + (1.187X_1_) + (0.837X_2_). ROC analysis showed that the area under the curve (AUC) of the model was 0.936. In the validation group, the AUC was 0.853.

**Conclusion:**

This study results provide a basis for precise prevention and control of multidrug-resistant tuberculosis bacteria in Nanning, help reduce the risk of transmission, and ensure public health safety of local and surrounding populations.

## 1 Introduction

Tuberculosis (TB) is an infectious disease caused by *Mycobacterium tuberculosis* (MTB). *Mycobacterium tuberculosis* can invade multiple organs in a patient’s body, but the primary site of infection is the lungs. *Mycobacterium tuberculosis* is one of the significant infectious pathogens globally. According to global tuberculosis reports, the number of TB patients worldwide increased by 3.7% in 2021 compared to 2020, reaching 450,000 cases, with China remaining one of the countries burdened with drug-resistant tuberculosis (1). In China, the incidence and mortality rates of tuberculosis are relatively high, with a significant proportion of drug-resistant patients. High drug resistance rate is a key factor in the spread of tuberculosis (2). As *Mycobacterium tuberculosis* continues to evolve to resist anti-tuberculosis drugs, the emergence of multidrug-resistant tuberculosis (MDR-TB) and extensively drug-resistant tuberculosis (XDR-TB) has occurred (3). Tuberculosis patients are mainly concentrated in rural areas, especially in economically underdeveloped regions in central and western China. The incidence of tuberculosis is higher among the floating population in the Nanning area, predominantly affecting young people with a wide range of activities, increasing the risk of tuberculosis transmission. Therefore, studying the factors influencing multidrug-resistant *Mycobacterium tuberculosis* in the Nanning area is of significant practical importance for formulating effective tuberculosis control strategies (4). However, there are currently no research reports on the molecular mechanisms and influencing factors of multidrug resistance in MTB isolates in the Nanning area. Existing studies primarily focus on macro-level data and have not sufficiently explored the molecular mechanisms and factors influencing multidrug resistance in MTB isolates from Nanning. This study addresses this gap. Furthermore, understanding multidrug resistance in regions like Nanning, and even Nanning, can provide crucial insights for the development of targeted global tuberculosis control strategies.

## 2 Research objects and methods

### 2.1 Research objects

This study retrospectively selected all sputum specimens from pulmonary tuberculosis patients collected at the Fourth People’s Hospital of Nanning from January 2021 to June 2022. Diagnostic criteria for pulmonary tuberculosis (5): The patient presents with typical symptoms, such as a cough and sputum production lasting for more than 2 weeks, along with low-grade fever and night sweats. Chest X-ray or CT imaging may reveal lung infiltrates, cavities, and other characteristic abnormalities. The presence of *Mycobacterium tuberculosis* can be confirmed through sputum smears or sputum culture. A diagnosis can be made if two or more of these criteria are met. All specimens were isolated, cultured, and identified to screen for clinically relevant strains. Cultures of the strains were performed with L-J medium, yielding a total of 346 initial specimens. After excluding duplicate strains from the same patient, non-*Mycobacterium tuberculosis* strains, and strains with missing drug susceptibility test results, a total of 337 *Mycobacterium tuberculosis* strains were included for analysis. To construct a predictive model, patients were divided into modeling group (*n* = 236) and validation group (*n* = 101) in a 7:3 ratio with a random number table method. In the modeling group, patients were further categorized into multidrug-resistant group (*n* = 47) and non-multidrug-resistant group (*n* = 189) based on drug susceptibility. First, assign numbers to all patients, ranging from 1 to 337. Then, select a starting point and direction from the random number table, and proceed by reading the numbers sequentially. Patients assigned numbers between 1 and 337 will be divided into two groups: the modeling group and the validation group, at a 7:3 ratio. Within the modeling group, further subdivision will occur based on whether the patients exhibit multi-drug resistance.

### 2.2 Research methods

#### 2.2.1 Relevant definitions

*Multidrug-resistant tuberculosis*: Tuberculosis caused by *Mycobacterium tuberculosis* strains that are resistant to at least isoniazid and rifampicin, two first-line anti-tuberculosis drugs, simultaneously.

*Treatment status*: Newly diagnosed tuberculosis patients refer to those who meet any of the following conditions: (1) Patients who have never received anti-tuberculosis treatment before. Patients who have started treatment according to standardized treatment regimens but have not completed the prescribed course of treatment. (2) Patients who have received non-standardized treatment regimens for less than 1 month. Retreated tuberculosis patients refer to those who meet any of the following conditions: (1) Patients who have received non-standard or irrational anti-tuberculosis treatment for a period not less than 1 month. (2) Patients who have failed initial treatment, experienced disease relapse, or returned for treatment due to other reasons.

*High-risk populations*: This includes individuals who have had close contact with cases, individuals infected with HIV/AIDS, diabetic patients, healthcare workers, prison staff, school and kindergarten staff, and patients with mental illness, among others.

#### 2.2.2 Drug susceptibility testing

Following the standards set by the World Health Organization/International Union Against Tuberculosis and Lung Disease, drug susceptibility testing of *Mycobacterium tuberculosis* was repeatedly conducted with the proportion method in this study. The experimental procedures strictly adhered to *Standardized Operating Procedures and Quality Assurance Manual for Drug Susceptibility Testing of Mycobacterium tuberculosis* (6). The drug concentrations used in the solid culture drug susceptibility testing were as follows: isoniazid 0.2 μg/mL, rifampicin 40 μg/mL, levofloxacin 2 μg/mL, and moxifloxacin 0.5 μg/mL. The MIC drug susceptibility concentrations were as follows: isoniazid 0.2 μg/mL (resistant), 0.4 μg/mL (resistant), 0.8 μg/mL (resistant), 1.6 μg/mL (resistant); rifampicin 0.25 μg/mL, 0.5 μg/mL, 1 μg/mL (moderately sensitive), 2 μg/mL (resistant); levofloxacin 1 μg/mL (moderately sensitive), 4 μg/mL (resistant); moxifloxacin 0.25 μg/mL (moderately sensitive), 0.5 μg/mL (resistant).

#### 2.2.3 Minimum inhibitory concentration (MIC) determination

Based on the phenotypic and molecular mutation characteristics of the isolated strains, they were divided into several groups, including isoniazid-resistant group, rifampicin-resistant group, levofloxacin-resistant group, moxifloxacin-resistant group, and fully sensitive group, with standard strains set as the control group. The strains were regrouped based on different mutation sites of drug-resistant genes, and drug susceptibility was determined with the minimum inhibitory concentration (MIC) method (MIC susceptibility plates provided by Zhuhai Encode Medical Engineering Co., Ltd.). For well-grown colonies after 3 weeks of culture, grinding, turbidity comparison, and bacterial suspension preparation were performed. The bacteria were then inoculated into microwell plates containing different concentrations of drugs, placed in a 37 °C incubator, and observed for colony growth weekly.

#### 2.2.4 Quality control

In each round of drug susceptibility testing, the standardized *Mycobacterium tuberculosis* strain H37Rv (ATCC 27294) was used as a sensitive control to validate the effective activity of the drugs on the drug susceptibility plates. Additionally, each drug susceptibility plate had two positive growth control wells without antibiotics. The test results were independently read by two experimenters and cross-checked to ensure the accuracy of the results.

### 2.3 Statistical analysis

The experimental data collected were analyzed with SPSS 27.0 (International Business Machines Corporation, Armonk, New York, United States). For normally distributed continuous data, X ± S was used for representation. Independent sample *t*-tests were employed for between-group comparisons, and *F*-test was used for multiple group comparisons. Count data were presented as frequencies or rates, and between-group comparisons were conducted with χ (2) test or Fisher’s exact test. Factors influencing the data were analyzed through univariate and binary logistics regression analysis, constructing predictive models with SPSS, and assessing the predictive value of the models through receiver operating characteristic (ROC) curve analysis. A significance level of *p* < 0.05 indicated statistical significance in differences.

## 3 Results

### 3.1 General information

Comparison of general data between the modeling group and the validation group of patients showed no statistically significant differences (*p* > 0.05); see Table 1 for details.

**Table 1:** Comparison of general data between modeling group and validation group of patients.

Baseline data	Classification	Modeling group (*n* = 236)	Validation group (*n* = 101)	*t/χ*^2^ value	*p*-value
Age (years)	<40	50	25	3.041	0.219
	40 ~ <60	69	28		
	≥60	157	48		
Gender	Male	179	79	0.221	0.638
	Female	57	22		
Treatment status	Initial Treatment	205	90	0.327	0.568
	Retreatment	31	11		
Residence	Urban	158	65	0.212	0.645
	Rural	78	36		
Occupation	Employed	115	50	0.017	0.896
	Unemployed	121	51		
Patient source	Direct visit	102	45	1.023	0.796
	Referral	28	12		
	Follow-up	67	24		
	Others	39	20		
High-risk population	Yes	68	30	0.027	0.869
	No	168	71		

### 3.2 Univariate analysis of influencing factors

Comparison of age, gender, residence, occupation, and patient source between two groups of patients in the modeling group showed no statistically significant differences (*p* > 0.05). However, there were statistically significant differences in the comparison of treatment status and high-risk population (*χ*^2^ =7.904, 5.401, *p* < 0.05), as shown in Table 2.

**Table 2:** Univariate analysis of influencing factors.

Baseline data	Classification	Multidrug-resistant group (*n* = 47)	Non-multidrug-resistant group (*n* = 189)	*t/χ*^2^ value	*p*-value
Age (years)	<40	10	40	2.407	0.300
	40 ~ <60	15	54		
	≥60	22	135		
Gender	Male	36	143	0.018	0.894
	Female	11	46		
Treatment status	Initial treatment	35	170	7.904	**0.005**
	Retreatment	12	19		
Residence	Urban	31	127	0.026	0.872
	Rural	16	62		
Occupation	Employed	25	90	0.468	0.494
	Unemployed	22	99		
Patient source	Direct visit	15	87	6.051	0.109
	Referral	5	23		
	Follow-up	20	47		
	Others	7	32		
High-risk population	Yes	20	48	5.401	**0.020**
	No	27	141		

Bolded p values indicate that the comparison between the two groups.

### 3.3 Binary logistics regression analysis

Treatment status and high-risk population were taken as independent variables and values are assigned to them, see Table 3 for details. Multidrug resistance was taken as the dependent variable (multidrug resistance = 1, non-multidrug resistance = 0) for analysis. The results of binary logistics regression analysis showed that treatment status and high - risk population were influencing factors of multidrug resistance (OR = 3.278, 2.308, *p* < 0.05), as shown in Table 4.

**Table 3:** Variable assignment.

Influencing factors	Assignment
Treatment status	Initial treatment = 0, retreatment = 1
High-risk population	No = 0, yes = 1

**Table 4:** Binary logistics regression analysis results.

Dependent variable	B	Standard error	Wald	*p*	Exp(B)	95%CI	
						Lower limit	Upper limit
Treatment status	1.187	0.422	7.921	0.005	3.278	1.434	7.494
High-risk population	0.837	0.348	5.795	0.016	2.308	1.168	4.562
Constant	−1.874	0.236	63.222	<0.001	0.154	–	–

### 3.4 Predictive model and ROC curve

**Figure 1:**
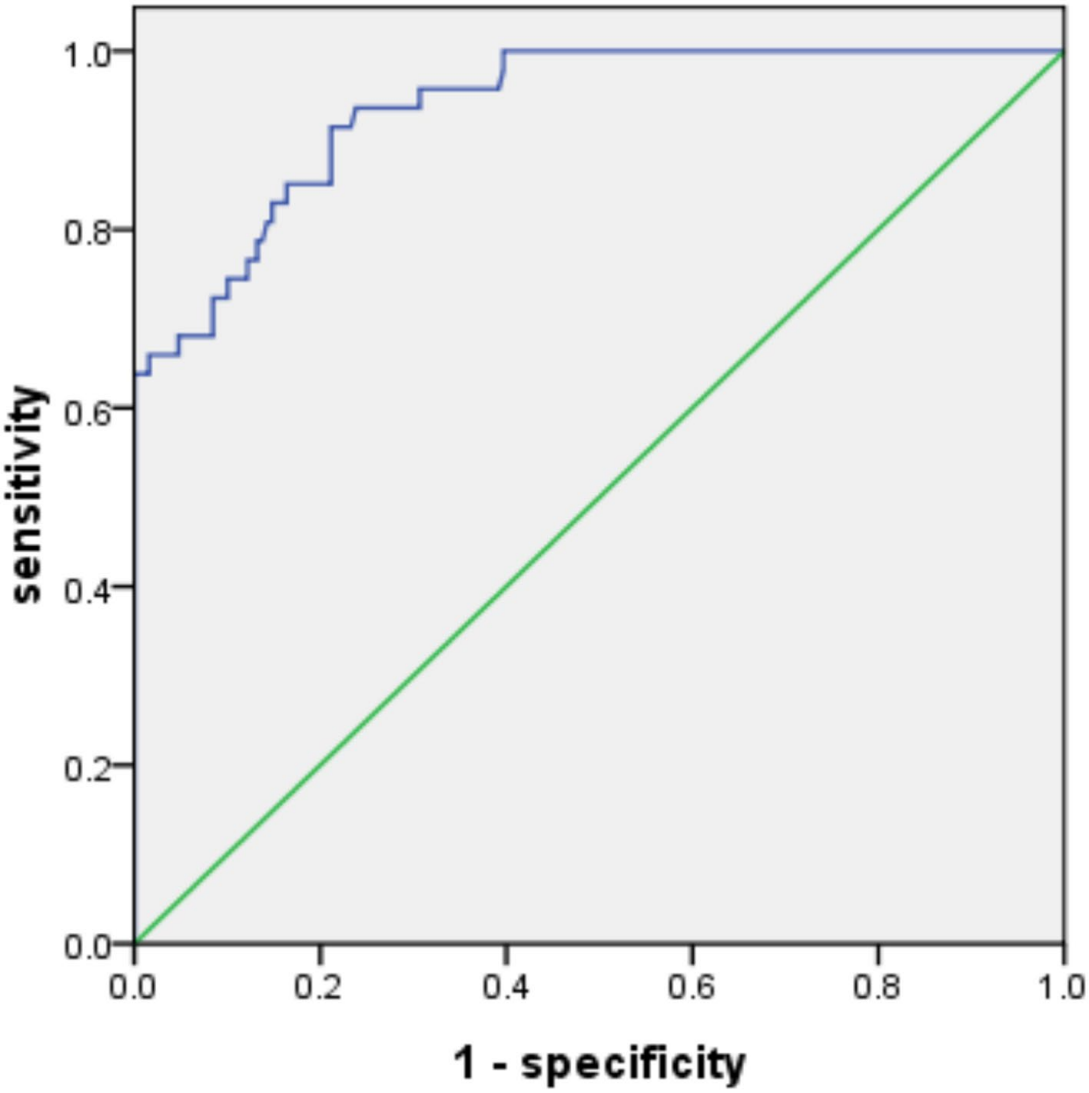
ROC curve of modeling group. The area under the ROC curve is close to 1, indicating that the model has excellent predictive performance in the modeling group.

**Figure 2:**
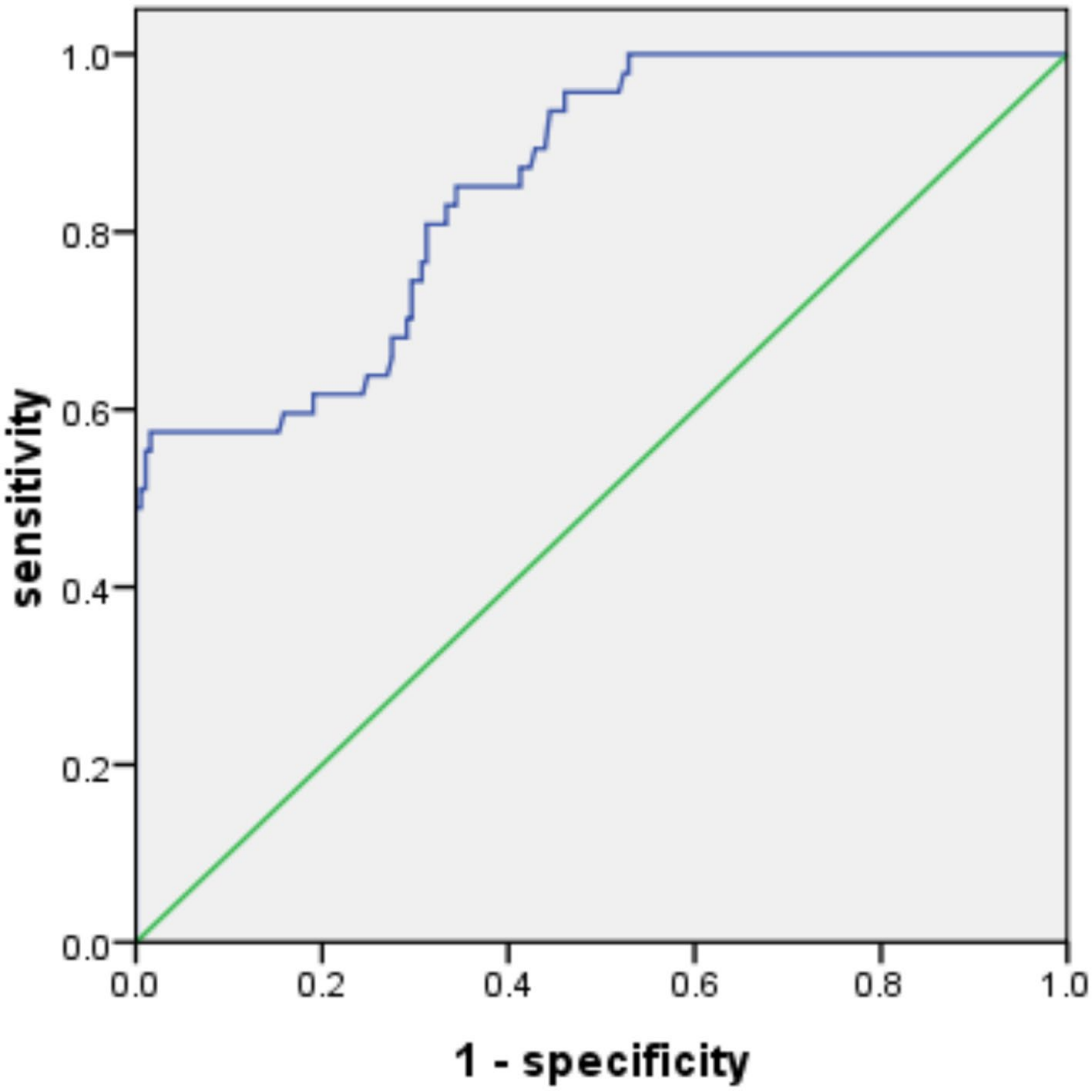
ROC curve of validation group. The ROC curve with 0.7 < AUC ≤ 0.9 indicates that the model has good predictive value in the validation group.

According to the results of logistics regression analysis, treatment status and high - risk population (named X_1_ and X_2_ respectively) were included in the constructed prediction model. The combined detection factor model expression was Logit(P) = −1.874 + (1.187X_1_) + (0.837X_2_). The ROC analysis results showed that the area under the prediction curve of the model (AUC) was 0.936 (95% CI: 0.902–0.970, *p* < 0.05), with a standard deviation of 0.017. The Youden index was 0.70, with a sensitivity of 91.50% and specificity of 78.84%, as shown in Figure 1. In the validation group, the area under the prediction curve of the model was 0.853 (95% CI: 0.795–0.911, *p* < 0.05), with a standard error of 0.029. The Youden index was 0.47, with a sensitivity of 57.45% and specificity of 89.42% at this time, see Figure 2 for details.

## 4 Discussion

With the gradual increase in the prevalence of tuberculosis in China, the high drug resistance rate of *Mycobacterium tuberculosis* poses a great challenge to the control of infectious diseases. The emergence of multidrug - resistant tuberculosis not only reduces the cure rate of patients, but also significantly increases the mortality rate (7). At present, the prevention and control situation of tuberculosis is still severe, and early identification of drug resistance is crucial for the management of tuberculosis patients. The culture-based drug susceptibility test is the gold standard for diagnosing drug-resistant tuberculosis, but this method takes a long time, affecting the timeliness of clinical treatment. Therefore, analyzing the influencing factors of drug resistance, especially focusing on factors that are helpful for timely control, is particularly important (8, 9).

In this study, through isolation culture and drug susceptibility testing, 47 strains of multidrug-resistant *Mycobacterium tuberculosis* were successfully identified, accounting for 13.95% of the total strains. Although this proportion is lower than some areas with high drug resistance prevalence, it still indicates that the prevention and control situation of multidrug - resistant tuberculosis in Nanning area is still severe and needs to be strengthened in monitoring and management. The study found that the treatment status of patients is one of the important factors affecting the multidrug resistance of *Mycobacterium tuberculosis*. Through the analysis of the treatment status of patients in the modeling group, it was found that there were significant differences in treatment history between the multidrug-resistant group and the non-multidrug-resistant group. Specifically, the proportion of retreatment in the multidrug - resistant group was higher. Analyzing the reasons, it may be closely related to the poor compliance and irregular medication of patients (10, 11). The samples in this study are primarily from Nanning, which may not fully capture the nationwide or global scope of retreatment cases for multi-drug resistant tuberculosis. Future research should aim to expand the sample size to enhance the generalizability of the findings.

In addition, this study found that high-risk populations is another important factor in the multidrug resistance of *Mycobacterium tuberculosis* in Nanning area. High-risk populations mainly include close contacts, HIV/AIDS infected individuals, and diabetic patients (12). Due to the common problems of low immunity, poor treatment compliance, and abnormal drug metabolism in these populations, they are more susceptible to infection with multidrug-resistant *Mycobacterium tuberculosis* or the development of drug resistance during treatment (13). Diabetic patients are more likely to develop drug resistance due to the decline in immune function caused by high blood sugar and the favorable environment for the proliferation of *Mycobacterium tuberculosis* (14, 15). In addition, diabetic drugs may interfere with the metabolism and excretion of anti-tuberculosis drugs, increasing the risk of drug resistance. A study by Rehman et al. (16) showed that diabetic patients have a higher risk of developing multidrug-resistant tuberculosis. HIV infected individuals, due to their compromised immune systems, have a significantly reduced ability to resist *Mycobacterium tuberculosis*, and HIV infection may accelerate the replication and spread of *Mycobacterium tuberculosis* (16, 17). The study also found that HIV infected individuals are at a greater risk of developing drug resistance during anti - tuberculosis treatment due to drug interactions and metabolic abnormalities. In this study, the HIV infection rate in the multidrug-resistant group was significantly higher than that in the non-multidrug-resistant group. Patients who have previously failed tuberculosis treatment may have drug-resistant mutant strains in their bodies. When they are re-infected with *Mycobacterium tuberculosis*, the drug - resistant mutant strains may become the dominant strains, leading to the emergence of drug resistance (18, 19). In this study, the proportion of previous treatment failure in the multidrug-resistant group was also significantly higher than that in the non-multidrug-resistant group (20, 21). Due to constraints in study time and resources, certain influencing factors could not be thoroughly examined, such as the underlying reasons for poor patient compliance, the susceptibility mechanisms of high-risk groups, and the detailed mutational processes of *Mycobacterium tuberculosis*. In addition, current studies have shown that exosome miRNAs, as “messengers” of inter-cellular communication, play a major role in inflammatory diseases. They can not only affect the inflammatory process by regulating inflammatory cell activation and factor release, but also have biomarker potential. It also provides new strategies for targeted therapy, and current research is promoting its systematic exploration and clinical transformation. Research related to Mycobacterium tuberculosis in this area can be increased in the future (22). These areas require further investigation. Therefore, future research should focus on a deeper exploration of the mechanisms underlying these factors and aim to minimize the impact of unknown variables, thereby enhancing the accuracy and reliability of the findings.

Through univariate and binary logistics regression analyses, this study identified treatment status and high-risk populations as the main influencing factors of multidrug-resistant *Mycobacterium tuberculosis* in Nanning area. Based on these factors, a combined detection factor model was constructed and validated with the receiver operating characteristic curve (ROC). The ROC analysis results showed that the area under the prediction curve of the model (AUC) was 0.936, with a standard error of 0.017 (95% CI: 0.902–0.970), and the Youden index was 0.70. At this time, the sensitivity was 91.50% and the specificity was 78.84%, indicating that the model had a high predictive value in the modeling group. In the validation group, the area under the prediction curve of the model was 0.853, with a standard error of 0.029 (95% CI: 0.795–0.911), and the Youden index was 0.47. At this time, the sensitivity was 57.45% and the specificity was 89.42%. Although the predictive value in the validation group was slightly lower than that in the modeling group, it still indicated that the model had good stability and generalization ability. The combined detection factor model provides a powerful tool for the prediction and prevention of multidrug-resistant tuberculosis in Nanning area. Through this model, clinicians can comprehensively assess the treatment status and characteristics of high-risk populations of patients, thereby predicting whether patients are likely to be infected with multidrug-resistant *Mycobacterium tuberculosis*. This helps clinicians to adjust treatment plans in a timely manner, strengthen monitoring and management of high-risk populations, thus reducing the incidence and transmission risk of multidrug-resistant tuberculosis (23). In the results of this study, the performance of the model between the modeling group and the verification group was significantly different, and there was a certain degree of overfitting. The main reasons were as follows. When modeling, it may rely too much on the specific characteristics and noise of the modeling group data and incorporate some accidental factors into the model, resulting in the model being too complex and refined and only suitable for the modeling group. However, there are differences in the data distribution and characteristics of the verification group and the modeling group, which makes the model unable to be effectively migrated. In addition, if the sample size of the modeling group is relatively insufficient, it will be difficult for the model to fully learn the general rules. When faced with new data, its generalization ability will be limited, and its effectiveness and sensitivity in the verification group will decrease.

Based on the findings of this study, Nanning urgently needs to enhance its drug resistance monitoring efforts. This includes establishing a robust drug resistance surveillance network, conducting regular monitoring, and accurately assessing the drug resistance profile of *Mycobacterium tuberculosis*. Such measures will provide a solid scientific foundation for the development and adjustment of prevention and control strategies.

Furthermore, treatment protocols should be optimized. Leveraging the data from drug resistance monitoring, treatment plans should be both evidence-based and flexible, avoiding single-drug therapies. Instead, combination therapies should be recommended, with appropriate drug dosages and treatment durations. In parallel, patient education and medication guidance must be strengthened to improve treatment adherence and ensure the successful implementation of treatment plans.

Currently, there is an urgent need to refine treatment strategies. This requires a shift away from monotherapy, favoring combination drug regimens, while ensuring both the proper dosage and treatment course. In addition, patient education should be prioritized to enhance treatment compliance. High-risk groups—such as the older adults, individuals with diabetes, HIV-positive patients, and those with a history of treatment failure—should be considered priority targets for management. These individuals require regular health check-ups and follow-up visits, early detection and intervention for multi-drug resistant tuberculosis, and reinforced health education to improve self-protection awareness.

Moreover, the introduction of new anti-tuberculosis medications, such as bedaquiline and delamanid, should be actively promoted to effectively reduce the incidence and transmission risk of multi-drug resistant tuberculosis. Finally, strengthening international collaboration is crucial to advance global efforts in tuberculosis prevention and control.

## 5 Conclusion

In conclusion, the multidrug resistance of *Mycobacterium tuberculosis* in Nanning area is becoming increasingly serious, posing a major challenge to tuberculosis prevention and control efforts. In response to this issue, it is crucial to take a series of effective prevention and control measures. Future research could explore the molecular mechanisms underlying drug resistance, shedding light on its genetic causes. Additionally, investigating patient behavior and treatment adherence would offer valuable insights, paving the way for new strategies to optimize prevention, control, and treatment approaches.

## Data Availability

The original contributions presented in the study are included in the article/supplementary material, further inquiries can be directed to the corresponding authors.
